# How pragmatic are the randomised trials used in recommendations for control of glycosylated haemoglobin levels in type 2 diabetic patients in general practice: an application of the PRECIS II tool

**DOI:** 10.1186/s13063-020-4215-5

**Published:** 2020-03-19

**Authors:** Isabelle Ettori-Ajasse, Elise Tatin, Gordon Forbes, Sandra Eldridge, Clarisse Dibao-Dina

**Affiliations:** 1 Département Universitaire de Médecine Générale, Faculté de Médecine, Université de Tours, EA 7505 – EES, 10 Boulevard Tonnellé, B.P. 3223, 37044 Tours cedex 1, France; 2 Université de Tours, Tours, France; 3 Kings College London, London, UK; 4 Barts & the London Queen Mary’s School of Medicine, London, UK; 5 Université de Tours, INSERM U1246 – SPHERE, Tours, France

**Keywords:** Pragmatic trial, Recommendations, General practice, Type II diabetes

## Abstract

**Background:**

Recommendations for good clinical practice have been reported to be difficult to apply in real life by primary care clinicians. This could be because the clinical trials at the origin of the guidelines are based on explanatory trials, conducted under ideal conditions not reflecting the reality of primary care, rather than pragmatic trials conducted under real-life conditions. The objective of this study was to evaluate how pragmatic are the clinical trials used to build the French High Authority of Health’s recommendations on the management of type II diabetes.

**Methods:**

Trials from the 2013 Cochrane meta-analysis that led to the 2013 French High Authority of Health’s recommendations on the management of type II diabetes were selected. Each trial was analysed by applying the PRECIS-2 tool to evaluate whether the trial was pragmatic or explanatory, according to the nine domains of PRECIS-2. Each domain was scored between 1 (very explanatory) and 5 (very pragmatic) by two blinded researchers, and consensus was reached with a third researcher in case of discrepancy. Median scores were calculated for each of the nine domains.

**Results:**

Twenty-three articles were analysed. Eight out of nine domains – namely eligibility, recruitment, setting, organisation, flexibility of delivery, flexibility of adherence, follow-up, and primary outcome – had a median score of less than 3, indicating a more explanatory design. Only the primary analysis domain had a score indicating a more pragmatic approach (median score of 4). In more than 25% of the articles, data to score the domains of recruitment, flexibility of delivery, flexibility of adherence, and primary analysis were missing.

**Conclusions:**

Trials used to build French recommendations for good clinical practice for the management of type 2 diabetes in primary care were more explanatory than pragmatic. Policy-makers should encourage the funding of pragmatic trials to evaluate the different strategies proposed for managing the patient’s treatment according to HbA1C levels and give clinicians feasible recommendations.

## Introduction

In France, recommendations to improve the management of patients with type II diabetes are based on the glycosylated haemoglobin (HbA1C) levels [1]. Practitioners have to adapt the patient’s treatment for them to achieve the recommended HbA1C levels according to their condition [1]. For most patients with type 2 diabetes, an HbA1c target less than or equal to 7% is recommended. For elderly or frail patients, the HbA1C target may be 8% or even 9% [1]. For newly diagnosed young and healthy patients, the HbA1C target is 6.5% [1].

These recommendations for managing the patient’s treatment according to HbA1C levels are based mainly on experts’ views of existing evidence. This evidence is often based on randomised trials. The latest guidelines are based on a 2013 Cochrane review that considered the effects of targeted intensive glycaemic control compared with conventional glycaemic control in patients with type 2 diabetes [2]. The review included randomised trials comparing mortality, macrovascular and microvascular complications and adverse events depending on predefined HbA1c targets [2]. However, there is a lack of studies comparing the different strategies, based on morbidity and mortality outcomes. Of the 45 recommendations, none is grade A (i.e., high quality of evidence, usually from well-performed randomised controlled trials), four are grade B (i.e., moderate quality of evidence, usually from randomised controlled trials with important limitations or very strong evidence from other designs) and 41 are grade C (i.e., low quality of evidence, usually from observational studies, clinical experience or controlled trials with serious flaws) [1].

However, an intensive strategy of management of HbA1C levels is not without risk: over a treatment period of five years, 117 to 150 patients would need to be treated to avoid one myocardial infarction and 32 to 142 patients to avoid one episode of microalbuminuria, whereas one severe episode of hypoglycaemia would occur for every 15 to 52 patients [3]. Severe hypoglycaemia was defined as a blood glucose level of less than 2.8 mmol/L (50 mg/dL) in patients with transient dysfunction of the central nervous system who were unable to treat themselves (requiring help from another person) [4].

For different reasons, practitioners do not follow recommendations [5]: lack of trust in the scientific basis of the recommendations, difficulties in applying the recommendations in real life, or for other reasons particular to the practitioner and their professional environment. The lack of trust in the scientific basis of the recommendations may come from the fact that the trials the recommendations are based on are not pragmatic, leading to results that cannot be applied in usual care.

A pragmatic trial is designed for testing the effectiveness of an intervention under real-world conditions, whereas an explanatory trial is designed for testing an intervention under ideal experimental conditions [6]. As mentioned by Godwin et al., “The explanatory trial seeks to maximise the internal validity by assuring rigorous control of all variables other than the intervention. The pragmatic trial seeks to maximise external validity to ensure that the results can be generalized” [7]. Trials are rarely wholly pragmatic or explanatory, and different elements of a trial may be more pragmatic or more explanatory. An assessment of how pragmatic or explanatory a trial is can be made by using PRECIS-2, a tool designed to evaluate whether a trial is more explanatory or pragmatic across nine different domains: eligibility criteria, recruitment, setting, organisation, flexibility (delivery), flexibility (adherence), follow-up, primary outcome, and primary analysis [8].

Therefore, we decided to determine whether the trials at the basis of the recommendations for the management of type II diabetes were more pragmatic or explanatory by using the PRECIS-2 tool. We decided to apply it to trials that were selected to build the recommendations on the management of type II diabetes on the basis of HbA1C levels [1]. Our aim was to determine whether those trials were more explanatory or pragmatic according to the PRECIS-2 tool.

## Methods

### Selection of randomised trials

Studies were eligible if they were randomised trials and included in the most recent Cochrane meta-analysis conducted on the topic that was cited in the recommendations and published in 2013 [2]_._ We restricted eligibility to the most recent Cochrane meta-analysis as recommendations were based on the results of the trials included in this review. Articles that reported studies that were not randomised trials or meta-analyses of randomised trials, articles reporting ancillary studies (i.e., studies derived from an original study), and articles written in a language other than English or French were excluded.

### Data collection

Each selected trial was analysed by using the PRECIS-2 tool [8]. Scores for each of the nine domains were from 1 (very explanatory) to 5 (very pragmatic) using a 5-point Likert scale [8]. A score of 3 was defined as equally pragmatic and explanatory [8]. In order to harmonise the scoring of the PRECIS-2 tool, the tool was tested on three randomly selected trials by the two researchers who would analyse all of the trials (ET and CD-D) and discussions on the scoring of the domains were carried out with all of the researchers (ET, CD-D, IE-A, SE, and GF) before the analysis of the remaining trials. The nine domains of the PRECIS-2 tool are described in Additional file 1: Appendix 1, and the result of the discussions on the scoring is detailed in Additional file 1: Appendix 2. Data were collected by two researchers (ET and CD-D) blinded from each other and using a standardised form developed by the researchers (CD-D, IE-A, SE, and GF). Collected data included the characteristics of the trials (details of the publication, design and main result) and scoring for the nine domains of the PRECIS-2 tool. In case of insufficient information in the selected article, the protocol of the article was consulted if it was referenced and published. After having collected the data, the two researchers (ET and CD-D) shared the results and discussed disagreeing scores to reach a consensus. If consensus between ET and CD-D could not be reached, the opinion of a third researcher (IE-A, SE or GF) was used to establish a consensus.

### Data analysis

The characteristics of the trials were analysed descriptively. The median score and interquartile range (Q1; Q3) over all trials were calculated for each domain in the PRECIS-2 tool. We also performed a descriptive analysis of the articles for which scoring needed a consensus between researchers and where there were missing data in the articles to score the domains of the PRECIS-2 tool.

## Results

### Selection of articles

**Fig. 1:**
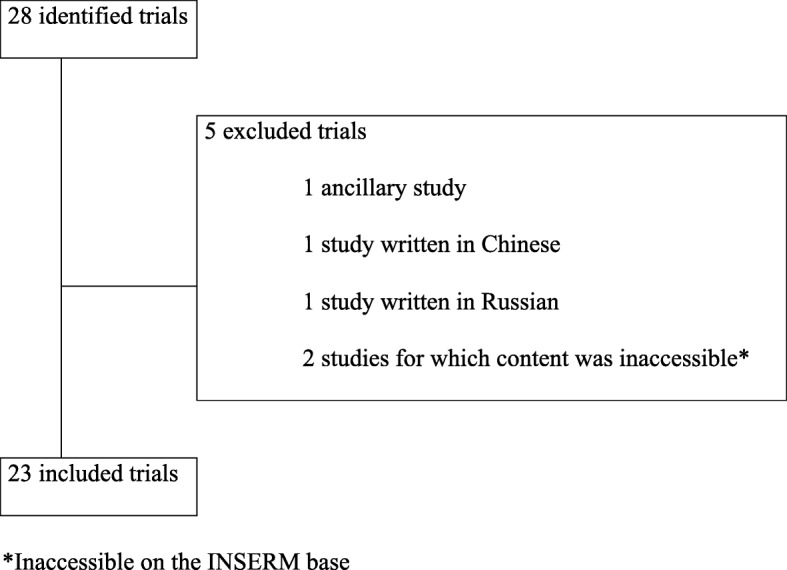
Flowchart of the selected trials

From the Cochrane meta-analysis published in 2013 on the HbA1C target-based therapeutic strategy of type 2 diabetes, 28 randomised clinical trials were identified [2]. Of these 28 randomised trials, 23 trials were included in the analysis (see Fig. 1).

### Characteristics of the included trials

The characteristics of the included trials are shown in Table 1. 2009 was chosen as a threshold date because it corresponds to the date of the first publication of the PRECIS tool [9]. All of the trials evaluated an intervention involving delivering a drug to achieve strict targets of HbA1C. The control group was a group with a usual care strategy or with less stringent blood glucose targets than the intervention group. The details of the intervention and primary criteria for each of the 23 trials are given in Additional file 1: Appendix 3. Of the 23 studies analysed, 17 were conducted in hospital settings.

**Table 1:** Characteristics of the 23 included trials

Characteristics		Number of articles	Missing data
		N (%)	N (%)
Publication date	Before 2009	14 (61)	0
	After 2009	9 (39)	
Country^a^	China	2 (9)	1 (4)
	US	5 (22)	
	UK	4 (17)	
	Denmark	3 (13)	
	Netherlands	2 (9)	
	Canada	2 (9)	
	Japan	1 (4)	
	New Zealand	1 (4)	
	Swiss	2 (9)	
	Finland/Norway	1 (4)	
	Greece	2 (9)	
Design	Two parallel groups	19 (83)	0
	Three parallel groups	3 (13)	
	Double factorial design 2 × 2	1 (4)	
Sample size median (Q1; Q3)		179 (82; 1068)	0
Number of centres	Monocentric studies	10 (45)	4 (17)
	Pluricentric studies	12 (55)	
Number of centres, median (Q1; Q3)		14 (1; 40)	
Follow-up in months, median (Q1; Q3)		51 (6; 67)	1 (4)

^a^The total number of countries cited exceeded the total number of trials as some trials were conducted in several countries

Scoring of trials according to the PRECIS-2 tool

The median scores for each domain in the PRECIS-2 tool are shown in Table 2.

**Table 2:** Median scores of the 23 trials for each domain of the PRECIS-2 tool

Title of domain	Median score	Number of articles with missing data	Articles for which scoring required a consensus	Articles for which scoring required a consensus between a score rather explanatory (<3) or pragmatic (>3)
	(Q1; Q3)	N (%)	N (%^a^)	N (%^a^)
Eligibility	2 (1; 2))	1/23 (4)	14/22 (64)	1/22 (5)
Recruitment	1 (1; 3)	10/23 (43)	10/13 (77)	5/13 (38)
Setting	2 (1; 2)	3/23 (13)	17/20 (85)	4/20 (20)
Organisation	2 (1; 2)	3/23 (13)	16/20 (80)	1/20 (5)
Flexibility of delivery	2 (2; 4)	6/23 (26)	13/17 (76)	1/17 (6)
Flexibility of adherence	2 (1.75; 2)	9/23 (39)	10/14 (71)	3/14 (21)
Follow up	2 (2; 3)	4/23 (17)	16/19 (84)	1/19 (5)
Primary outcome	2 (1; 3.25)	3/23 (13)	12/20 (60)	5/20 (25)
Primary analysis	4 (1; 5)	6/23 (26)	12/17 (71)	0/17 (0)

^a^The consensus was any discrepancy in the scores between the two researchers (ET and CD-D)

Eight out of nine PRECIS-2 domains had a median score of less than 3. “Recruitment”, “Flexibility of adherence” and “Primary outcome” were the hardest domains for reaching the consensus between explanatory and pragmatic.

**Fig. 2:**
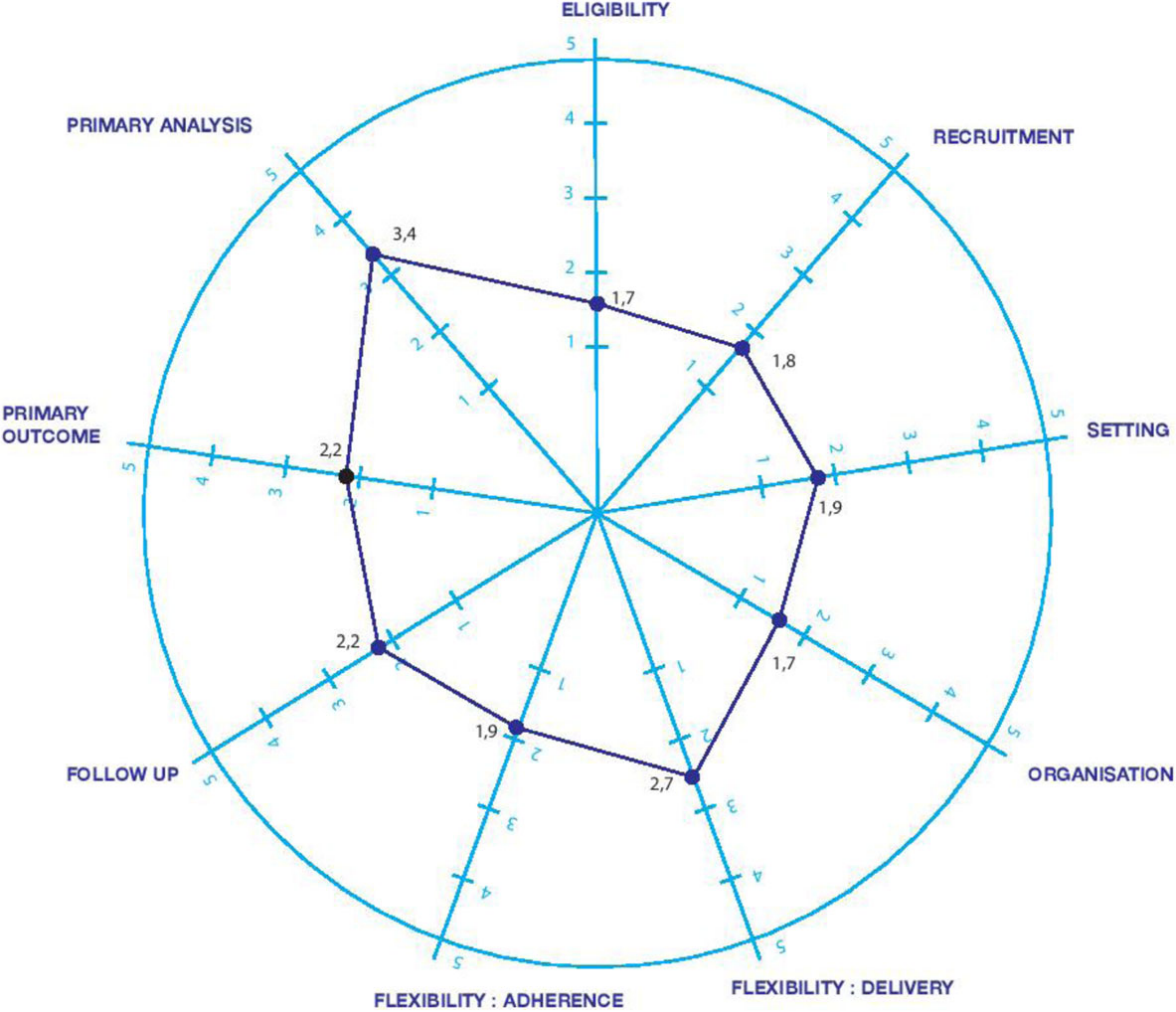
Graphical representation of the PRECIS-2 wheel

The results were graphically represented with the “wheel” of PRECIS-2 in Fig. 2.

The details of the median scores before and after consensus are available in Additional file 1: Appendix 4.

For every PRECIS-2 domain, between 60% and 85% of the articles required a consensus, mainly on the degree to which the trial was pragmatic (between the scores 4 or 5) or explanatory (between scores 1 or 2). Disagreements between pragmatic score (>3) and explanatory scores (<3) occurred in between 0% and 22% of articles, depending on which domain was considered.

## Discussion

### Summary of results

We found that clinical trials contributing to recommendations on the therapeutic strategy of type 2 diabetes were more explanatory than pragmatic. In fact, only the domain on primary analysis was more pragmatic with a median score greater than 3 (4). The lack of information reported in the article to score its explanatory or pragmatic aspect concerned mainly recruitment, flexibility of delivery, flexibility of adherence and primary analysis, and more than 25% of articles had insufficient information. The majority of the scoring has required a consensus to define the degree to which the trial was pragmatic or explanatory.

### Interpretation of results

For eligibility, the selection of the population was more explanatory than pragmatic. Most trials evaluated the intervention under optimal conditions and not with populations that would present in primary care. For example, patients with comorbidities that could interfere with study outcomes, patients living too far from the study site, or patients who were unable to manage insulin and cope with hypoglycaemia were excluded [10–13]. These highly selective samples excluded patients usually encountered in general practice, which may be one of the barriers to implementing recommendations in usual care. Furthermore, some studies included very specific populations, such as veterans, that were not comparable with the typical French diabetic population [14].

Most of the trials were carried out in hospital settings, leading to the scores for domains of setting, recruitment, organisation, primary outcome and flexibility of delivery being more explanatory than pragmatic. If recommendations of good clinical practice were addressed to the management of hospitalised patients with diabetes, those results would have been more pragmatic in that context. However, some characteristics of the organisation or intervention that are provided in the hospital setting would not be available in primary care, such as involvement of specialised staff to administer the treatment and educate the patient or more intensive monitoring of the patient [15–21]. In the hospital, the treatments were administered by nurses, which ensured regular intake and reduced the risk of non-compliance. On the other hand, the outpatient was autonomous, and there was no guarantee of compliance with the treatment prescribed by the general practitioner. For the primary outcome, the median score of 2 in favour of a rather explanatory methodological choice was because either the primary outcome was not observable in general practice or it required the intervention of specialists [20, 22, 23]. For instance, the study by Natarajan et al. used the change of volume of intimal hyperplasia within the stent as primary outcome [22].

The domain on the primary analysis was the only one out of the nine with a median score greater than 3, suggesting that the majority of trials were more pragmatic in relation to this domain. This was because most of the studies analysed the results following the intention-to-treat principle [24]. This sort of analysis is recommended for intervention’s regulatory approval and in CONSORT (Consolidated Standards of Reporting Trials) guidelines.

### Strengths and limitations

Our study was the first from the perspective of the French primary care setting to use a graphic tool to illustrate that the trials on which recommendations for good clinical practice were based were not pragmatic. We chose to use the PRECIS-2 tool because it had already been used successfully for a systematic review in order to assess whether the pragmatism of the trials was a source of heterogeneity in the trial results [25]. As a prevention for a desirability bias according to our initial hypothesis of an excess of explanatory trials, the researchers had different backgrounds: an experienced general practitioner (CD-D) and a medical student (ET) independently scored the trials, whereas consensus was reached thanks to a third general practitioner (IE-A) and two biostatisticians (SE and GF) who studied the applicability of the PRECIS-2 tool [26]. However, in our study, many scores were finalised only after discussion between researchers. Some areas of the PRECIS-2 tool were subject to interpretation despite the examples given in the article by Loudon et al. [8]. This can be explained by the purpose for which the PRECIS-2 tool was created, namely the evaluation of protocols intended for researchers and not the evaluation of the published trials. To be applicable for the latter objective would require a greater precision in the way of scoring each domain, to ensure a better homogeneity of the evaluations of the trials.

There was also a lot of missing data in the articles to score certain domains of PRECIS-2. However, missing data were less frequent for domains that were detailed in the CONSORT guidelines for reporting randomised trials [24].

### Implications

According to our results, the clinical trials on which recommendations for good clinical practice were based had primarily explanatory features, which could constitute an obstacle to the application of recommendations to the therapeutic strategy of type 2 diabetes in general practice. However, we cannot be sure that the lack of pragmatism of the evidence of the guidelines is the only reason why general practitioners do not follow the guidelines. This can contribute to this lack of trust, but other controversies around the evidence might also play a role: in particular, the Cochrane review concluded that there was no benefit of the intensive glycaemic control on mortality compared with the conventional glycaemic control, and French guidelines still recommended an HbA1C targets less than 7% in most of the cases.

Ideally, both types of trials would be required: explanatory trials to demonstrate the efficacy of interventions under ideal conditions and pragmatic trials performed under the usual conditions of practice to improve the applicability of the results in general practice. This requires the involvement of general practitioners and their patients in clinical research and also methodological and organisational adaptations to integrate research into daily care practice.

## Conclusions

Our study has highlighted the fact that the clinical trials leading to recommendations on the therapeutic strategy of type 2 diabetes were more explanatory than pragmatic. Diabetes researchers could concentrate on more pragmatic trials. Policy-makers should encourage the funding of pragmatic trials to evaluate the different strategies proposed in the recommendations for managing the patient’s treatment according to HbA1C levels.

## Supplementary information

**Additional file 1: Appendix 1.** The nine domains of the PRECIS-2 tool. **Appendix 2.** Consensus on the scoring of the domains of the PRECIS-2 tool. **Appendix 3.** Intervention, control and primary outcome of the 23 included trials. **Appendix 4.** Median scores before and after consensus.

## Data Availability

All of the dataset is available on request.

## Abbreviations

CONSORT: Consolidated Standards of Reporting Trials
HbA1C: Glycosylated haemoglobin
PRECIS-2: Pragmatic-explanatory continuum indicator summary 2nd version
