# Supplementary material for: How pragmatic are the randomised trials used in recommendations for control of glycosylated haemoglobin levels in type 2 diabetic patients in general practice: an application of the PRECIS II tool
Source: Trials. 2020 Mar 19;21:281. doi: 10.1186/s13063-020-4215-5 (PMC7081519; doi:10.1186/s13063-020-4215-5)
Supplement: Supplementary file 1 — Additional file 1: Appendix 1. The nine domains of the PRECIS-2 tool. Appendix 2. Consensus on the scoring of the domains of the PRECIS-2 tool. Appendix 3. Intervention, control and primary outcome of the 23 included trials. Appendix 4. Median scores before and after consensus. [file 13063_2020_4215_MOESM1_ESM.docx]

Appendix 1. The 9 domains of the PRECIS-2 tool


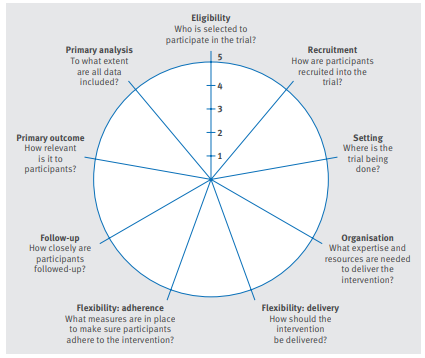


A blank PRECIS-2 wheel. Reproduced with permission from Treweek Shaun (permission will be asked to BMJ in case of article acceptance).

PRECIS-2 is a tool to evaluate if a trial is more explanatory or pragmatic. Nine domains are evaluated and scored between 1 (explanatory) and 5 (pragmatic).

1. Eligibility—To what extent are the participants in the trial similar to those who would receive this intervention if it was part of usual care?

2. Recruitment—How much extra effort is made to recruit participants over and above what would be used in the usual care setting to engage with patients?

3. Setting—How different are the settings of the trial from the usual care setting?

4. Organisation—How different are the resources, provider expertise, and the organisation of care delivery in the intervention arm of the trial from those available in usual care?

5. Flexibility (delivery)—How different is the flexibility in how the intervention is delivered and the flexibility anticipated in usual care?

6. Flexibility (adherence)—How different is the flexibility in how participants are monitored and encouraged to adhere to the intervention from the flexibility anticipated in usual care?

7. Follow-up—How different is the intensity of measurement and follow-up of participants in the trial from the typical follow-up in usual care?

8. Primary outcome—To what extent is the trial’s primary outcome directly relevant to participants?

9. Primary analysis—To what extent are all data included in the analysis of the primary outcome?

Loudon K, Treweek S, Sullivan F et al. The PRECIS-2 tool: designing trials that are fit for purpose .BMJ 2015;350 (2147):1-11

Appendix 2. Consensus on the scoring of the domains of the PRECIS-2 tool

For domains for which we did not have data to determine whether the trial was more explanatory or pragmatic (according to the criteria/examples from the article referenced n°4), we decided to assign the NE (No Evaluable) rating after consensus following independent readings. This choice was preferred rather than a score of 3 on which we initially opted; indeed, score 3 corresponded to an interpretation of the trial data between pragmatic and explanatory according to PRECIS-2 and not a lack of data or a lack of precision. Missing data with a NE was not taken into account in the calculation of the average scores.

For the "primary outcome" domain, when it was a surrogate outcome frequently used in practice (eg HbA1c) or a clinical criterion with the intervention of an adjudication committee, we scored 3. The "surrogate outcome" was indeed considered as a more explanatory than pragmatic element in the PRECIS-2 tool. For example, in the Jaber study, the surrogate outcome was HbA1c. This criterion remained insufficient to evaluate the effectiveness of the care provided by professionals on the management of diabetes and the prevention of its complications.

For the "adherence" item, as specified in PRECIS-2, the item was considered as NA (not applicable) for intravenous injection as an intervention.

Appendix 3. Intervention, control and primary outcome of the 23 included trials

Appendix 4. Median scores before and after consensus

|  | Median score before consensus (CDD)  Median (Q1;Q3) | Median score before consensus (ET)  Median (Q1;Q3) | Median score after consensus  (CDD, ET, IEA, SE, GF)  Median (Q1;Q3) |
| --- | --- | --- | --- |
| Eligibility | 2 (1;2) | 2 (2;3) | 2 (1;2) |
| Recruitment | 1 (1;2) | 4 (2;4) | 1 (1;3) |
| Setting | 1 (1;3) | 2 (2;4) | 2 (1;2) |
| Organisation | 2 (1;2) | 2 (2;2) | 2 (1;2) |
| Flexibility delivery | 2 (1;2.25) | 3 (2;4) | 2 (2;4) |
| Flexibility adherence | 2 (1;2.5) | 2 (2;2) | 2 (1.75;2) |
| Following | 2 (1;2) | 3 (2;3) | 2 (2;3) |
| Primary outcome | 2 (1;3) | 4 (1;4) | 2 (1;3.25) |
| Primary analysis | 4.5 (2;5) | 5 (3.5;5) | 4 (1;5) |
